# Skeletal muscle oxidative adaptations following localized heat therapy

**DOI:** 10.1007/s00421-023-05159-7

**Published:** 2023-03-23

**Authors:** Mohammed Ihsan, Mariem Labidi, Sebastien Racinais

**Affiliations:** 1 Research and Scientific Support, Aspetar Orthopedic and Sports Medicine Hospital, Doha, Qatar; 2 Education Department, Aspetar Orthopedic and Sports Medicine Hospital, Doha, Qatar; 3 Faculty of Sport Sciences and Physical Education, CETAPS, University of Rouen, Mont-Saint-Aignan, France

**Keywords:** Heat stress, Near-infrared spectroscopy, Human skeletal muscle, Microvascular function, Mitochondrial function

## Abstract

Repeated heat treatment has been shown to induce oxidative adaptations in cell cultures and rodents, but similar work within human models is scarce. This study investigated the effects of 6 weeks of localized heat therapy on near-infrared spectroscopy-(NIRS) derived indices of muscle oxidative and microvascular function. Twelve physically active participants (8 males and 4 females, age: 34.9 ± 5.9 years, stature: 175 ± 7 cm, body mass: 76.7 ± 13.3 kg) undertook a 6-week intervention, where adhesive heat pads were applied for 8 h/day, 5 days/week, on one calf of each participant, while the contralateral leg acted as control. Prior to and following the intervention, the microvascular function was assessed using NIRS-based methods, where 5 min of popliteal artery occlusion was applied, and the reperfusion (i.e., re-saturation rate, re-saturation amplitude, and hyperemic response) was monitored for 2 min upon release. Participants also performed a 1-min isometric contraction of the plantar flexors (30% maximal voluntary contraction), following which a further 2 min interval was undertaken for the assessment of recovery kinetics. A 20-min time interval was allowed before the assessment protocol was repeated on the contralateral leg. Repeated localized heating of the gastrocnemius did not influence any of the NIRS-derive indices of microvascular or oxidative function (*p* > 0.05) following 6 weeks of treatment. Our findings indicate that localized heating via the use of adhesive heat pads may not be a potent stimulus for muscle adaptations in physically active humans.

## Introduction

Improvements in the oxidative potential of skeletal muscle are associated with enhanced endurance performance (Fitts et al. 1975) and a reduction in risk factors for various chronic diseases (Bishop-Bailey 2013; Pedersen and Saltin 2015). Increased mitochondrial content and function, as well as increased capillarization, are the established adaptations underpinning whole-body metabolic health and performance (Hawley et al. 2014). It is well recognized that endurance exercise is the most potent stimulus to enhance such adaptations. However, this approach may be inappropriate for several patient groups limited by immobilization or reduced physical activity. Therefore, alternative interventions are warranted to preserve muscle oxidative function in both sporting and clinical situations (e.g., injury, illness, and immobilization).

Passive heat therapy has emerged as a promising intervention shown to improve or avert the decline in muscle force, contractile function, mitochondrial and vascular adaptations among healthy (Racinais et al. 2017; Ihsan et al. 2020a; Sabapathy et al. 2021) and immobilized humans (Hafen et al. 2018, 2019). Heat therapy modalities have been either whole body or localized treatment. Whole-body heat treatment is generally administered for 40–60 min through hot water immersion (Brunt et al. 2016; Sabapathy et al. 2021) or via climatic chambers/hot room (Racinais et al. 2017; Hesketh et al. 2019; Ihsan et al. 2020a), with 6–8 weeks (3–5 sessions/week) of treatment shown to improve macrovascular function and structure (Brunt et al. 2016), as well as muscle capillarization (Hesketh et al. 2019). However, mitochondrial muscle density was not influenced (Hesketh et al. 2019). Localized heat therapy may be more suitable for some populations who may be intolerant of the severe heat stress conferred by whole-body modalities or in populations where isolated muscle groups are targeted due to immobilization or injury. In support, localized heat therapy has been shown to maintain mitochondrial function and attenuate muscle atrophy in immobilized humans (Hafen et al. 2019). Such modalities include the use of water-perfused garments (Kuhlenhoelter et al. 2016; Kim et al. 2020) or pulsed-wave diathermy (Hafen et al. 2018, 2019).

Commercially available adhesive heat-generating pads are a simple method, available to most people and do not require extensive procedures or expensive equipment. Despite the relative ease of this heat therapy method, there is minimal research examining the effectiveness of this modality on various adaptive responses. Only two studies have investigated the use of such heat pads, with one reporting an increase in muscle mass and strength (Goto et al. 2011), while another reporting no changes in similar variables (Labidi et al. 2020) following 10 weeks (8 h/day, 4 days/week) and 6 weeks (8 h/day, 5 days/week) of localized heat treatment, respectively. However, it is unknown if localized treatments via heat pads are sufficient to impact muscle microvascular and aerobic function to enable appropriate use of such therapy in clinical/sport practice.

Near-infrared spectroscopy (NIRS) is a common method to assess changes in muscle oxygenation. Muscle re-oxygenation rates following submaximal exercise has been shown to associate with the activity of muscle oxidative enzymes (Puente-Maestu et al. 2003). Moreover, the reperfusion response following arterial occlusion demonstrates significant associations with flow-mediated dilation measurements, rendering this technique as an appropriate measure of microvascular function (McLay et al. 2016a, b, c). As such, NIRS-derived measures may offer a simple, non-invasive method to assess changes in muscle oxidative and microvascular function following heat therapy. The purpose of this study was to investigate the changes in NIRS-derived indices of muscle oxidative and microvascular function. It is hypothesized that 6 weeks of localized heat therapy will improve muscle oxidative and microvascular function in healthy humans.

## Methods

### Participants

Twelve physically active participants (8 males and 4 females, age: 34.9 ± 5.9 years, stature: 175 ± 7 cm, body mass: 76.7 ± 13.3 kg) took part in this study. All participants were part of a larger companion study published elsewhere (Labidi et al. 2020). They regularly engaged in aerobic exercise and played team sport (~ 90 min over 2–3 sessions/week), but were not undertaking any structured exercise program. Participants refrained from all exercise, as well as alcohol and caffeine for at least 24 h prior to the experimental sessions. All requirements and risks associated with the study were communicated to the participants, and written informed consent was obtained prior to participation. The study was approved by the scientific committee of the hospital (CMO/0000167/ak) and by an external ethics committee (ADL-Q, E2017000255).

### Experimental design and procedures

All participants attended a familiarization session 1 week prior, and 2 experimental testing sessions separated by 6 weeks of localized heat therapy. Heat pads were applied for 8 h/day and 5d/week on one lower leg, while the contralateral leg acted as control. Control (CON) and heated (HEAT) legs were determined in a counterbalanced fashion based on the strength measures obtained during the familiarization session.

#### Familiarization

Participants were accustomed to the experimental procedures during familiarization. Following sufficient recovery (~ 5–10 min), participants performed 3-maximal voluntary isometric contraction (MVC) of the plantar flexors on an isokinetic dynamometer (Biodex system 3, Shirely, NY, USA). Contractions were performed with participants in a supine position, with full extension of the hips and knees, and arms crossed in front of the chest. One foot was positioned on the foot adapter connected to the head of the dynamometer and secured with two straps. The axis of rotation of the lever arm was aligned with the axis of rotation of the ankle. Each contraction lasted 5 s and was separated by a 1-min recovery period. The test/retest correlation for this measure was 0.93 (Labidi et al. 2020). A 5-min recovery period was allowed before performing the MVCs on the contralateral leg. The torque data were collected at 2000 Hz using MP35 hardware (Biopac Systems, Santa Barbra, CA) and specific software (BSL Pro Version 3.6.7, Biopac Systems).

#### Heat therapy

Two adhesive heat pads (The Heat Company, Altenmarkt, Austria) were placed on the gastrocnemius muscle from 8:00 am to 4:00 pm, for 5 days a week, for 6 consecutive weeks. The intervention duration was based on a previous study utilizing a similar mode of heat therapy (i.e., heat pads), where an increase in several genes associated with vascular morphogenesis were evident (8 h/day, 4 days/week for 10 weeks) (Goto et al. 2011). The gastrocnemius was targeted as ankle injuries account for almost a quarter of all injuries in high-school athletes (Nelson et al. 2007), leading to substantial atrophy within this muscle during the first week of immobilization (Stevens et al. 2004). The heat pads were applied directly on the skin, and further secured with an elasticated tubular bandage. Each heat pad measured 9 × 13 cm and was positioned abreast covering the medial and lateral gastrocnemius regions. The heat pads are air-activated and generate heat through the exothermic reaction involving the oxidation of iron powders/filings. Preliminary work (*n* = 5) demonstrated peak muscle temperatures (at 2 cm depth) of 37.3 ± 0.9 °C and 37.6 ± 1.0 °C (from 33.5 ± 0.8 °C) following 3 and 6 h of application, respectively (Labidi et al. 2020). The pads were removed 48 h before testing.

#### Near-infrared spectroscopy and testing

Microvascular and muscle oxidative function was assessed using a wireless, continuous-wave NIRS system (Portamon; Artinis Medical Systems, BV, Netherlands) via measurement of tissue saturation index [TSI (%)]. The Portamon unit consisted of three emitter diodes positioned 30, 35, and 40 mm from the detector, and emitted infrared light at wavelengths of 760 and 850 nm. NIRS signal penetration depth is estimated to be 17.5 mm.

The Portamon unit was affixed to the medial gastrocnemius muscle, along the largest circumference observed within the calf. The probe area was measured and recorded relative to the vertical and horizontal distance from the popliteal fold and from the vertical axis along the calf, respectively for accurate repositioning during POST. The Portamon unit was secured using black adhesive tape reinforced with elasticated bandages to prevent movement and signal contamination from external light sources. Calibration was up-to-date in our Portamon system, and the measurement quality of the TSI signal (i.e., the TSI fit factor) was ≥ 99% as per manufacturers’ recommendation prior to all experimental sessions. Before probe placement, the area of investigation was assessed for subcutaneous tissue thickness using skinfold calipers (Harpenden, British Indicators Ltd, UK).

Once ready, participants assumed a supine position on the isokinetic dynamometer, with hip and knee fully extended. A pressure cuff (SC12D, Hokanson, Bellevue, USA) connected to a rapid inflator (Hokanson, Bellevue, USA) was attached to the participants’ quadriceps just above the knee articulation. Baseline measurements were undertaken following a 10-min period to allow for NIRS signals to stabilize, throughout which participants lay rested in the supine position. Arterial occlusion (300 mmHg) was then applied for 5 min, following which, 2 min of post-occlusion monitoring was undertaken. Assessment of recovery kinetics was adapted from previous work (Ryan et al. 2013). Participants then performed a 1-min isometric contraction of the plantar flexor equivalent to 30% of their individual MVC determined during familiarization, following which a further 2 min interval was undertaken for the assessment of recovery kinetics. Identical absolute intensities were utilized during the post-treatment test following 6 weeks. Contraction force was controlled by visual feedback, where a laptop screen showing changes in force was displayed in front of the participants.

A 20-min time interval was allowed before the assessment protocol was repeated on the contralateral leg. Movement was allowed during this period, but participants assumed the testing position at least 10 min prior to the commencement of the assessment. All NIRS data were collected at 10 Hz using dedicated software (Oxysoft, Artinis Medical Systems) and down sampled to 1 Hz for further analyses.

#### NIRS data analysis

**Fig. 1:**
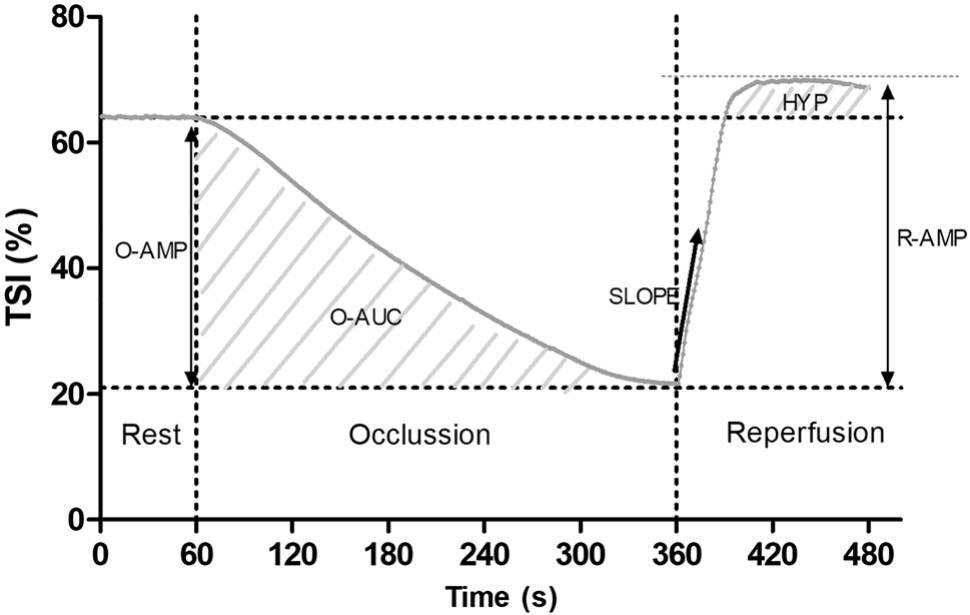
Typical changes in TSI profile in a representative participant during 5 min of arterial occlusion and subsequent recovery. *O-AMP* maximal change in TSI amplitude during arterial occlusion, *O-AUC* area under the curve during arterial occlusion, *SLOPE* TSI reperfusion rate following cuff release, *R-AMP* maximal change in TSI amplitude during reperfusion, *HYP* hyperaemic response determined by the area under the curve following cuff release above resting values

**Fig. 2:**
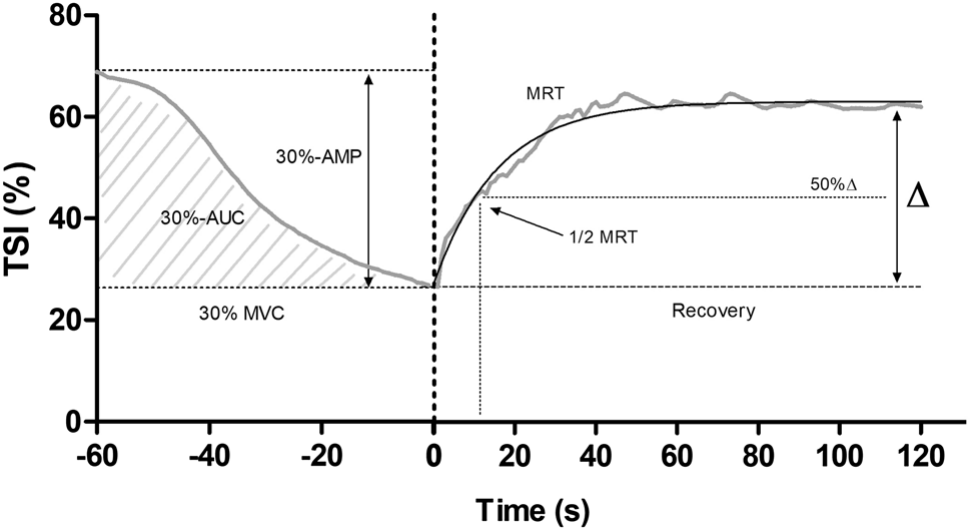
Typical changes in TSI profile in a representative participant during 1 min of isometric contraction of the plantar flexors (30% MVC) and subsequent recovery. *30%-AUC* area under the curve during isometric plantar flexion, *30%-AMP* maximal change in TSI amplitude during isometric plantar flexion

Representative TSI changes during arterial occlusion and reperfusion, and during 30% MVC and recovery are presented in Figs. 1 and 2, respectively. Amplitude (O-AMP; difference between average rest value and the min value achieved at the end of arterial occlusion) and the area under the curve (O-AUC) during occlusion were taken as indices of the occlusion stimulus (McLay et al. 2016b). The reperfusion rate (SLOPE) was determined on the initial 10 s window following cuff release and taken as an index of microvascular function (McLay et al. 2016a, b). The intraday and interday coefficient of variation for this measure has been reported to be 9 ± 4% and 14 ± 5%, respectively (McLay et al. 2016c). SLOPE was determined using a linear function (Ihsan et al. 2020b): TSI = *a* × *t* + *b*, where *a* is the slope (%·s^−1^), *t* is the time (s) and *b* is the *y*-intercept (%). The reperfusion amplitude (R-AMP; difference between the maximum and minimum TSI values achieved at the end of arterial occlusion and following cuff release, respectively) and hyperemic response (HYP; AUC following cuff release above resting values) were taken as indices of post-occlusive reactive hyperemia (Martin et al. 2009; Lacroix et al. 2012).

During 30% MVC, changes in TSI amplitude (30%-AMP; difference between the maximum and minimum TSI values during 30% MVC) and AUC during the contraction (30%-AUC) were determined to quantify the metabolic stimulus. TSI recovery kinetics following 30% MVC was modeled using a mono-exponential function: y = end + *∆* x e^−1/*Tc*^, where y represents TSI, end is the TSI value at the end of 30% MVC, *∆* is the change in TSI from end exercise to recovery and *Tc* is the fitting time constant. The mean response time (MRT) represents the adaptation of 63% of the overall TSI response (∆), while ½ MRT was determined by calculating the time taken to achieve 50% *∆*. Owing to poor fit to the model (i.e., *r*^2^ < 0.6), the analysis on recovery kinetics was limited to *n* = 7.

### Statistics

Data distribution was assessed using the Shapiro–Wilk test, which demonstrated no deviations from normality for all variables. Assumption of sphericity was assessed by Mauchly’s test of sphericity, and the Greenhouse–Geisser correction was applied where this assumption was violated. All NIRS-derived indices were analyzed a two-way repeated measures ANOVA (condition × time). Statistical significance was accepted at *p* ≤ 0.05. Partial eta square (Ƞ_p_^2^) was used to assess effect size with Ƞ_p_^2^ > 0.01, Ƞ_p_^2^ > 0.06 and Ƞ_p_^2^ > 0.14 indicating small, medium and large effects, respectively. All data are presented in mean ± SD and all statistical analysis was performed using SPSS version 19 (IBM SPSS, Chicago, IL).

## Results

Changes in NIRS-derived indices of microvascular function are presented in Table 1. O-AUC and O-AMP were similar over time and between conditions (*p* > 0.05). No main effects for time, condition, or interaction were noted in R-AMP, HYP, and SLOPE, indicating no benefit following 6 weeks of localized heat therapy on NIRS-derived indices of microvascular function in human gastrocnemius muscle.

**Table 1:** Changes (mean ± SD) in NIRS-derived indices of microvascular function following 6 weeks of localized heat therapy (*n* = 12)

	CON		HEAT		Main Effects (Ƞ_p_^2^)		
	PRE	POST	PRE	POST	Time	Condition	Interaction
O-AUC (A.U)	2730 ± 1400	3142 ± 1075	2707 ± 983	2743 ± 978	0.388 (0.068)	0.495 (0.043)	0.180 (0.157)
O-AMP (%)	26 ± 13	27 ± 8	26 ± 12	26 ± 8	0.696 (0.014)	0.727 (0.012)	0.600 (0.026)
R-AMP (%)	32 ± 15	34 ± 10	33 ± 16	35 ± 11	0.589 (0.027)	0.808 (0.006)	0.871 (0.003)
HYP (%)	562 ± 310	608 ± 243	621 ± 401	707 ± 344	0.452 (0.052)	0.144 (0.184)	0.648 (0.020)
SLOPE (%·s^−1^)	1.3 ± 0.5	1.2 ± 0.5	1.3 ± 0.5	1.3 ± 0.5	0.632 (0.022)	0.828 (0.005)	0.543 (0.035)

*O-AUC* area under curve during arterial occlusion, *O-AMP* occlusion amplitude, *R-AMP* recovery amplitude, *HYP* hyperaemic response, *SLOPE* rate of TSI recovery following occlusion release

*p* values for ANOVA main effects are presented with partial eta square (Ƞ_p_^2^) in brackets

Changes in NIRS-derived indices of muscle oxidative function are presented in Table 2. The area under the curve (30%-AUC) and TSI amplitude (30%-AMP) during sustained plantar flexion exercise (30% MVC) were similar over time and between conditions Likewise, no main effects for time, condition, or interaction were noted in MRT and 1/2 MRT.

**Table 2:** Changes (mean ± SD) in NIRS-derived indices of muscle oxidative function following 6 weeks of localized heat therapy (*n* = 7)

	CON		HEAT		Main effects		
	PRE	POST	PRE	POST	Time	Condition	Interaction
30%-AUC (A.U)	687 ± 381	707 ± 305	695 ± 288	694 ± 274	0.886 (0.004)	0.985 (< 0.001)	0.883 (0.004)
30%-AMP (%)	30 ± 15	29 ± 12	32 ± 16	33 ± 13	0.923 (0.002)	0.198 (0.258)	0.420 (0.111)
MRT (s)	19.7 ± 6.1	22.4 ± 7.2	20.1 ± 4.2	17.9 ± 6.6	0.773 (0.015)	0.438 (0.103)	0.143 (0.322)
1/2 MRT (s)	13.6 ± 4.2	16.0 ± 4.8	14.5 ± 3.5	12.4 ± 4.6	0.869 (0.005)	0.498 (0.080)	0.056 (0.482)

*30%-AUC* area under curve during 30% isometric MVC, *30%-AMP* amplitude during 30% isometric MVC, *MRT* mean response time, *1/2 MRT* 50% mean response time

*p* values for ANOVA main effects are presented with partial eta square (Ƞ_p_^2^) in brackets

## Discussion

This study investigated the changes in NIRS-derived indices of muscle oxidative and microvascular function following 6 weeks of localized heat therapy. Our findings did not show any changes in any NIRS-derived indices (Tables 1, 2), hence suggest that heat therapy may not confer additional benefits in individuals who are physically active or training.

 Following extensive work in rodents demonstrating beneficial effects of heat on skeletal muscle health and function, similar research in humans is emerging, with a variety of heat-based modalities able to offer either whole body or localized treatments. We limited the current investigation to commercially available heat-generating pads, given the relative ease of this modality can be used to target specific muscle groups and incorporated into rehabilitative programs (Ihsan et al. 2019). The current study reports no beneficial effects following 6 weeks of heat therapy on gastrocnemius muscle oxidative or microvascular function. Our findings are in contrast with the previous works investigating the effects of localized heat therapy on human mitochondrial and angiogenic signaling/adaptations (Kuhlenhoelter et al. 2016; Hafen et al. 2018). Specifically, using pulsed-wave diathermy or water-perfused sleeves, acute localized heating of the quadriceps lasting 90–120 min has been shown to upregulate mitochondrial-related signaling and the expression of angiogenic factors (Kuhlenhoelter et al. 2016; Hafen et al. 2018). Moreover, short-term repeated quadricep heat treatment for 6 consecutive days (120 min/session) has been shown to increase the expression of electron transport chain proteins and mitochondrial function (Hafen et al. 2018). Longer term quadriceps heat treatment spanning 8 weeks (90 min sessions × 5 days/week) has been shown to increase the expression of muscle angiogenic factors and averted the temporal decline in capillarization in type 2 fibers, but did not influence the mitochondrial content (Kim et al. 2020).

The current findings are likely influenced by the muscle temperatures achieved by the heat pad modality. As reported in a companion paper (Labidi et al. 2020), the current heat pad increased the gastrocnemius muscle (~ 2 cm depth) temperature from 33.5 ± 0.8 °C to 37.3 ± 0.9 °C and 37.6 ± 1.0 °C following 3 h and 6 h of heat pad application, respectively. Such temperatures are likely insufficient to confer meaningful adaptations within our participant cohort. Indeed, increased activation of regulatory kinases was observed at temperatures above 39 °C (Yoshihara et al. 2013) in rodents exposed to varying degrees of heat stress (37–41 °C), Likewise in humans, enhanced mitochondrial-centered signaling/adaptations were observed when localized treatments elevated quadriceps muscle temperatures to ~ 40 °C (Hafen et al. 2018), but not ~ 38 °C (Ihsan et al. 2020a). Interestingly, Goto et al. (2011) reported increased quadriceps cross-sectional area and knee extensor strength following 10 weeks of quadriceps heat pad application, albeit achieving slightly higher muscle temperatures (38.2–38.3 °C at 1.5 cm depth) compared with the current study (37.3–37.6 °C at 2 cm depth). We are unsure how to reconcile these differences in findings, although it is important to note that the treatment time (10 vs. 6 weeks), investigated muscle groups (quadriceps vs. calf), and participant characteristics (sedentary vs. physically active) were different between Goto et al. (2011) and the current study.

Differences in participant characteristics, along with changes in their physical activity levels during the study period may have also contributed towards the differential findings observed. Specifically, Hafen et al. (2018) recruited sedentary participants, while Kim et al. (2020) reported a marked decrease in participants’ habitual exercise routines due to their involvement in the study. The decrease in physical activity coupled with increased sedentary time likely accounts for the decline in capillarization, which was successfully averted by including heat treatment (Kim et al. 2020). In contrast, participants in the current study were physically active in endurance or team sport, and maintained their weekly physical activity levels throughout the study period. Indeed, the data presented in a separate companion paper demonstrated no temporal changes in muscle mass or strength in the control limb that received no heat treatment (Labidi et al. 2020). Likewise, the current study demonstrates no temporal changes in all NIRS-derived indices in the control limb following the 6-week study period. This perhaps indicates that heat treatment may be of most benefit in dilapidating conditions such as muscle damage (Kim et al. 2019; Sabapathy et al. 2021) or where the mechanical stimulus is reduced or limited (Hafen et al. 2019). In support of this notion, localized heat treatment via pulsed-waved diathermy attenuated the decline in mitochondrial and macrovascular function, as well as muscle mass during 10 days of unilateral lower-limb immobilization (Hafen et al. 2019; Hyldahl et al. 2021). Conversely, no additional benefit was observed when quadriceps heat application was supplemented during and following training sessions for 12 weeks (2–3 sessions/week) of lower body resistance training (Stadnyk et al. 2017). Regardless, the authors acknowledge that some studies have demonstrated improved muscle adaptations following repeated heat treatment in healthy physically active participants (Racinais et al. 2017), albeit using whole-body heat therapy. As such, further research is needed to elucidate the interaction between contractile activity and heat stimulus to best formulate heat therapy for appropriate populations.

The duration of heating was based on the previous work utilizing a similar mode of heat therapy, where an increase in several genes associated with vascular morphogenesis were evident following 10 weeks of quadriceps heat application (8 h/day, 4 days/week) (Goto et al. 2011). We are also confident of the NIRS technique, and demonstrate that the metabolic stimuli during occlusion (i.e., O-AUC and O-AMP) and sustained plantar flexion (30%-AUC and 30%-AMP) were consistent between CON and HEAT legs prior to and following 6 weeks of treatment.

In summary, no beneficial effect was observed following 6 weeks of localized heat therapy on changes in gastrocnemius aerobic function. In trained/active individuals, localized treatments may likely require a more potent heat stimulus resulting in higher muscle temperatures or whole-body heating modalities. Given that the application of heat therapy to modulate skeletal muscle aerobic function is still in its infancy, future studies should determine the optimal balance between heating modalities, duration, intensity, and frequency of treatment to confer meaningful adaptations in different populations.

## Data Availability

The raw data supporting the conclusions of this article will be made available by the authors, without undue reservation, to any qualified researcher.

## Abbreviations

NIRS: Near-infrared spectroscopy
MVC: Maximal voluntary contraction
TSI: Tissue saturation index
O-AMP: Occlusion amplitude
O-AUC: Occlusion area under the curve
R-AMP: Reperfusion amplitude
HYP: Hyperemic response
MRT: Mean response time
ANOVA: Analysis of varance
